# Multivariate Analysis of the Failure of Removal of the Urinary Catheter within 48 Hours after Transurethral Enucleation and Resection of the Prostate

**DOI:** 10.1155/2020/8241637

**Published:** 2020-02-13

**Authors:** Yukun Wu, Binshen Chen, Chunxiao Liu

**Affiliations:** Department of Urology, Zhujiang Hospital, Southern Medical University, No. 253, Gongye Avenue, Guangzhou, Guangdong 510282, China

## Abstract

**Objective:**

To assess the value of clinically relevant data for predicting the failure of removal of the urinary catheter within 48 hours after TUERP. *Materials and Methods*. We retrospectively analyzed the medical records of 357 patients who underwent TUERP between January 2015 and July 2018, all of whom stopped bladder irrigation and removed urinary catheter within 48 hours after the operation. According to whether the removal of the catheter was successful, the patients were classified into 2 groups: Group A was successful and group B was a failure. Univariate analysis was performed to determine the association between the failure of removal of the catheter and the patients' preoperative clinical characteristics. Logistic regression analysis and receiver operating characteristic analysis (ROC) were conducted to establish the prediction model. Then the area under the curve (AUC) and the cut-off value were calculated.

**Results:**

357 patients were divided into group A (*n* = 305, 85.4%) and group B (*n* = 305, 85.4%) and group B (*P*=0.006), history of acute urinary retention (AUR) (*P*=0.006), history of acute urinary retention (AUR) (*P*=0.006), history of acute urinary retention (AUR) (*P*=0.006), history of acute urinary retention (AUR) (*P*=0.006), history of acute urinary retention (AUR) (*P*=0.006), history of acute urinary retention (AUR) (*P*=0.006), history of acute urinary retention (AUR) (*P*=0.006), history of acute urinary retention (AUR) (*P*=0.006), history of acute urinary retention (AUR) (

**Conclusion:**

This study demonstrated that IPSS, QoL, drug medication, history of AUR, TPV, and IPP are independent factors associated with the failure of removal of the urethral catheter within 48 hours after TUERP.

## 1. Introduction

Benign prostatic hyperplasia (BPH), which causes lower urinary tract symptoms (LUTS), is a common diagnosis among the aging male population with increasing prevalence [1, 2]. It is a histologic diagnosis characterized by enlargement of the prostate gland from a nonmalignant proliferation of glandular and stromal elements in the transition zone of the prostate [3]. The development of BPH in males is an age-related process. Histologic evidence of BPH is identified in approximately 20% of men in their 40 s, in 50% to 60% of men in their 60 s, and in up to 90% of men in their 70 s and 80 s [3, 4]. Transurethral Resection of the Prostate (TURP) is the historical gold standard therapy for LUTS secondary to obstruction from BPH [5]. However, it is not a standard anatomical surgery; postoperative complications are still there; for example, it is usually recommended for prostates less than 80 mL [6], postoperative BPH recurrence [7]. In addition, the whole resection process is the repeated opening of the perforator vessels of the prostate, so the treatment of the blood-rich prostate is limited [8–10]. Other complications such as TURP-syndrome and bladder neck contracture were also high [11]. There are a number of clinical studies suggesting that TUERP and TURP have similar efficacy, but TUERP removes hyperplastic glands more thoroughly, with shorter operative time, less bleeding, and shorter bladder irrigation and catheterization time [12, 13]. The catheterization time varies from study to study [14–17]. This article investigates the risk factors for the failure of removal of the urinary catheter within 48 hours and conducts correlation analysis.

## 2. Materials and Methods

### 2.1. Patients

We retrospectively analyzed the medical data of 357 patients who underwent TUERP between January 2015 and March 2018. Inclusion criteria were male patients ≥18 years, and/or failed medical therapy, recurrent urinary tract infections, and/or recurrent episodes of urinary retention. Exclusion criteria were previous urethral/prostatic surgery, known prostate cancer or urethral strictures, and urodynamically diagnosed neurogenic bladder, and known bladder stones, bladder diverticulum, or bladder tumor [18, 19]. The urinary catheter was removed within 48 hours.

### 2.2. Surgical Methods

Under general or spinal anesthesia the patient was placed in the lithotomy position. The TUERP procedure was performed using a Plasma Kinetic resectoscope (27 F) with 160 W cutting power and 80 W coagulation power, or diode laser system was set at 120 W. The procedure was performed as follows. The ureteral orifices, bladder neck, and verumontanum were identified. The incision was begun close to the verumontanum from the 5 to the 7 o'clock positions, and the urethral mucosa was incised deeply to the level of the surgical capsule, A network of vessels runs on the inner surface of the surgical capsule and sends perforating vessels to the prostatic adenoma. The middle lobe, left lobe, and right lobe were dissected off the surgical capsule in a retrograde fashion from the apex toward the bladder. The adenoma was dissected from the capsule plane. The blood vessels to the adenoma were coagulated at the time of dissection. When the whole adenoma was almost dissected from the capsule, then they were resected into smaller prostatic chips. At the end of both procedures, a 22Fr triple-lumen catheter was inserted into the bladder and irrigation was initiated [20, 21].

### 2.3. Medical Records

All patients underwent a general and urologic preoperative evaluation, including age, BMI, history of AUR, IPSS, QoL, TPV, IPP, RUV, PSA, drinking, smoking, hypertension, diabetes, drug medication, urine culture, and surgical methods.

### 2.4. Statistical Analysis

These variables were statistically investigated to identify any factors that might result in the failure of the removal of the catheter. Mann–Whitney's *U* test for continuous variables and the *χ*^2^ test for categorical variables were applied to identify which preoperative variables are significant. Then multiple logistic regression analysis was performed to select relevant variables, and a *P* value < 0.05 was considered significant. Finally, the ROC curve was plotted to calculate the predictive value and find the optimal cut-off points of preoperative variables associated with the persistence of postoperative storage symptoms. All statistical analyses were performed using SPSS 22.0.

## 3. Results

Of the 357 patients enrolled in the study, 305 (85.4%) had successful urinary catheter removal within 48 hours (group A) and 52 (14.6%) failed (group B). The preoperative clinical characteristics are shown in Table 1. There are significant differences in drug medication (*P*=0.006), history of AUR (*P* ≤ 0.001), smoking (*P*=0.045), IPSS (*P* ≤ 0.001), IPP (*P*=0.006), PSA (*P*=0.047), RUV (*P* ≤ 0.001), QoL (*P* ≤ 0.001), and TPV (*P*=0.043) between the two groups; however, there is no significant difference in surgical methods (*P*=0.923), hypertension (*P*=0.181), diabetes (*P*=0.0916), drinking (*P*=0.437), urine culture (*P*=0.305), BMI (*P*=0.817), age (*P*=0.770), creatinine (Cr) (*P*=0.294), or operation time (*P*=0.931).

Multivariate analysis revealed that drug medication, history of AUR, IPSS, IPP, QoL, and TPV significantly associated with the failure of the removal of the catheter (Table 2). Smoking, PSA, and RUV were excluded from the multivariate analysis.

**Figure 1:**
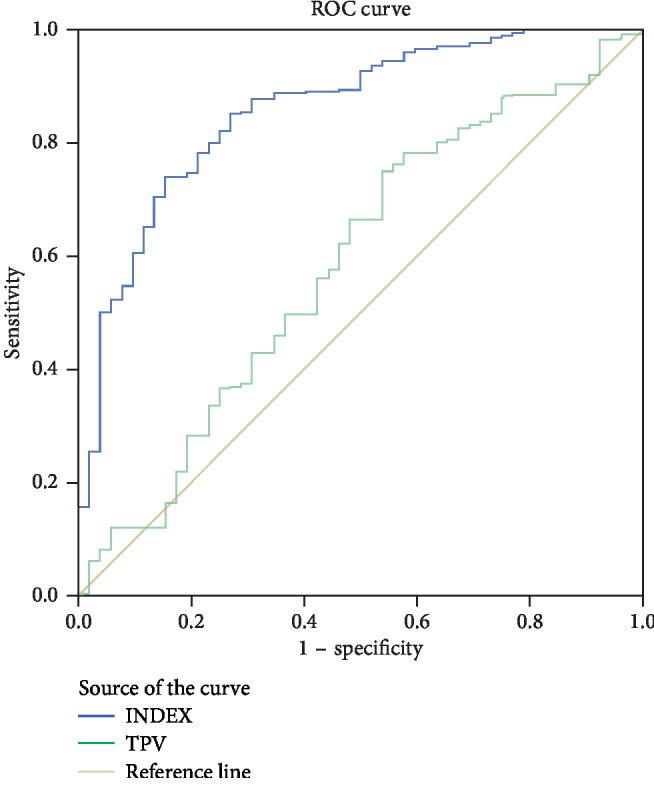
ROC curves to determine the cut-off values, sensitivity, and specificity of the variables.

A predictive model using logistic regression to determine the failure of the removal of the catheter was defined as follows: INDEX = 10.862 − 1.376 × (IPSS) − 1.185 × (QoL) − 1.062 × (drug medication) + 1.079 × (history of AUR) + 0.030 × (TPV) − 0.867 × (IPP) with area under the curve of 0.860 obtained from the ROC curve analysis. The predictive model had a cut-off value of 1.7725, and the sensitivity for predicting the failure of removal of the urethra was 74.1% and the specificity was 84.6%. The ROC curves of preoperative variables significantly related to the catheter are shown in Figure 1. The AUC, cut-off value, sensitivity, and specificity of these variables alone are shown in Table 3.

## 4. Discussion

BPH is a disease with slow progress. The prostate gland gradually increases and then presses the urethra, resulting in the patient's dysuria, which seriously affects daily life [22]. Treatment for BPH should be based on a comprehensive consideration of the patient's age, symptoms, prostate volume, economic conditions, health status, and other factors. As a first-line treatment for treatment selection, drug therapy is indeed effective and safe for some elderly patients, but long-term use will bring great economic pressure to patients [23, 24]. With the aging of the population structure, the number of patients is increasing year by year. When prostate hyperplasia develops to a certain extent, urinary incontinence, dysuria, and other symptoms may occur. The commonly used first-line treatment in men with symptomatic BPH is the association of a-adrenergic blockers with 5a-reductase inhibitors. Nonresponders and patients with drug intolerance may require surgical procedures [25, 26].

Although TURP is still considered to be the “gold standard” for surgical treatment of BPH [27, 28], the procedure is not suitable for large prostate and is associated with intraoperative and postoperative hemorrhage, TUR syndrome, postoperative urethral stricture, postoperative recurrence, and other complications [8]. Therefore, safe, minimally invasive, and effective surgical methods have become an important topic in urology.

TUERP combined with the concept of conventional open surgery and transurethral resection of the prostate, can not only completely remove the gland but can also reduce bleeding; it is less invasive to the body and has been recognized by clinical experts [29]. Although the energies used for enucleation of the prostate by urologists may be different, such as holmium laser [30], thulium laser [31], diode laser [32], or plasma kinetic energy [14, 33], the principle is similar which include identification of the tissue plane between the adenoma and the surgical capsule, and anatomical enucleation of the adenoma, and this technique has the advantage of being an extravesical procedure, better controlling the bleeding and avoiding bladder incision. TUERP can reduce wound repair time, thus shortening bladder irrigation time, catheterization time, and postoperative hospital stay [12, 34].

There is no uniform conclusion for the time of catheterization time after TUERP. The successful removal of the urethra after surgery is affected by many factors. In order to remove the urinary catheter safely and effectively at the early stage, and to avoid the need to reindwell the urinary catheter, this study combines clinical data with multiple factors to establish a predictive model, improves the success rate of removing the catheter within 48 hours after surgery, and tries to avoid repeated indwelling of the catheter after surgery.

In our study, 52 patients (14.6%) needed to reindwell the urinary catheter, and it showed that the prostate volume of group A (55.43 ± 44.16) was larger than that of group B (42.31 ± 42.54), and the difference was statistically significant (*P* < 0.05). Prostate volume was an independent risk factor for predicting the failure of catheter removal within 48 hours. We can notice that the smaller prostate has higher PSA, maybe it is because the inflammation of the prostate is more severe. The failure of removing the small prostate's urinary catheter within 48 hours may be caused by congestion and edema of the prostate. The urethra is squeezed and it takes a long time to recover. If the catheter is removed early, the risk of failure is higher. At the same time, drug medication before the surgery is also an independent risk factor, so if patients take medication before surgery, it is safer to remove the catheter within 48 hours after surgery. There is a significant difference between the patients with and without the acute urinary retention history (*P* < 0.05), and the OR value was 2.943 (95% CI 1.024–8.459). It was suggested that the existence of a history of urinary retention was an independent risk factor for catheter removal within 48 hours after operation, and patients with a history of urinary retention were 2.943 times more likely to fail to remove the catheter. Other clinical indicators, such as PSA, although statistically significant between group A and group B, could not be used as a valid predictor of failure to remove the catheter in multivariate logistic regression analysis.

This study comprehensively used the predictive value of each clinical data to establish a model to improve the success rate of the removal of the urinary catheter within 48 hours. The diagnostic model has a cut-off value of 1.7725. The sensitivity and specificity of this predictive model is 74.1% and 84.6%, respectively. This model takes into account diagnostic sensitivity and specificity. It can improve the success rate of removing the catheter within 48 hours, and at the same time, reduce or prevent the patient from indwelling catheterization again.

Our research also had some limitations: (1) this study is a single-center retrospective analysis, and the results still need to be verified by a prospective randomized multicenter study and (2) the lack of some valuable urodynamic data, such as the extent of detrusor overactivity and urine flow rate.

## 5. Conclusion

In clinical practice, the combination of various preoperative variables can predict the failure of removal of the catheter within 48 hours in this study. Our results suggest that various preoperative variables with cutoffs are available for the prediction. And further studies are required to test the reproducibility of the data.

## Figures and Tables

**Table 1:** Preoperative clinical characteristics of enrolled patients.

	Group A (*n* = 305)	Group B (*n* = 52)	*P*
Age (years)	70.00 ± 8.98	71.00 ± 7.67	0.770
PSA (ng/L)	6.20 ± 5.69	9.03 ± 8.44	0.047^*∗*^
Cr (*μ*mol/L)	87.00 ± 52.75	85.50 ± 55.46	0.294
Surgery time (min)	70.00 ± 32.66	72.50 ± 27.42	0.931
PVR (219)	20.00 ± 183.80 (179)	70.00 ± 177.33 (40)	≤0.001
QoL	5 ± 1.47	6 ± 0.693	≤0.001^*∗*^
TPV (mL)	55.43 ± 44.16	42.31 ± 42.54	0.043
Drug medication	162 (53.1%)	17 (32.7%)	0.006^*∗*^
History of AUR	82 (26.9%)	27 (51.9%)	≤0.001^*∗*^
Surgical methods			
Bipolar	215 (70.5%)	37 (71.2%)	0.923
Laser	90 (29.5%)	15 (28.8%)	
Hypertension	128 (42.0%)	27 (51.9%)	0.181
Diabetes	80 (26.2%)	14 (26.9%)	0.916
Smoking	108 (35.4%)	26 (50.0%)	0.045^*∗*^
Drinking	129 (42.3%)	25 (48.1%)	0.437
IPSS			
Asymptomatic (0)	0 (0.0%)	0 (0.0%)	≤0.001^∗^
Mildly symptomatic (1 ≤ IPSS ≤ 7)	24 (7.9%)	0 (0.0%)	
Moderately symptomatic (8 ≤ IPSS ≤ 19)	134 (43.9%)	12 (23.1%)	
Severely symptomatic (20 ≤ IPSS ≤ 35)	147 (48.2%)	40 (76.9%)	
Urine WBC			
−	160 (52.5%)	22 (42.3%)	0.305
+/−	26 (8.5%)	7 (13.5%)	
+	119 (39.0%)	23 (44.2%)	
BMI			
Underweight (BMI ≤ 18.49)	22 (7.2%)	3 (5.8%)	0.817
Normal weight (18.5 ≤ BMI ≤ 24.99)	200 (65.6%)	35 (67.3%)	
Overweight (25.0 ≤ BMI ≤ 29.99)	77 (25.2%)	12 (23.1%)	
Obesity (30 ≤ BMI)	6 (2.0%)	2 (3.8%)	
IPP (mm)			
Grade I (0 ≤ IPP ≤ 4.9)	193 (62.3%)	19 (36.5%)	≤0.001^*∗*^
GradeII (5 ≤ IPP ≤ 9.9)	23 (7.5%)	2 (3.9%)	
Grade III (10 ≤ IPP)	89 (29.2%)	31 (59.6%)	

Values are presented as median ± standard deviation. ^*∗*^Significant values; *P* < 0.05.

**Table 2:** Multiple regression analysis of clinical variables.

Variables	B	S.E.	*P*	Exp (B)	95% C.I. for EXP (B)	
					Lower	Upper
IPSS	−1.376	0.503	0.006^*∗*^	0.253	0.094	0.678
QoL	−1.185	0.326	≤0.001^*∗*^	0.306	0.161	0.579
Drug medication	−1.062	0.471	0.024^*∗*^	0.346	0.137	0.870
History of AUR	1.079	.0539	0.045^*∗*^	2.943	1.024	8.459
TPV	0.030	0.009	0.001^*∗*^	1.030	1.012	1.049
IPP	−0.867	0.290	0.003^*∗*^	0.420	0.238	0.742
Constant	10.862	2.642	≤0.001^*∗*^			

B: regression coefficient; CI: confidence interval; Exp (B): odds ratio; SE: standard error; ^*∗*^significant values; *P* < 0.05.

**Table 3:** AUC, cut-off value, sensitivity, specificity, PPV, and NPV of each variable.

Variables	AUC	Cut-off value	Sensitivity (%)	Specificity (%)
INDEX	0.860	1.7725	74.1	84.6
TPV	0.588	37.03	75.1	46.2

## Data Availability

The data used to support the findings of this study are included within the article.

## Abbreviations

TUERP: Transurethral enucleation and resection of the prostate
BMI: Body Mass Index
AUR: Acute urinary retention
IPSS: International prostate symptom score
QoL: Quality of life score
TPV: Total prostate volume
IPP: Intravesical prostatic protrusion
ROC: Receiver operating characteristic
PSA: Prostate-specific antigen
AUC: Area under the curve
BPH: Benign prostatic hyperplasia
LUTS: Lower urinary tract symptoms
TURP: Transurethral resection of the prostate
RUV: Residual urine volume.

## References

[1] Sarma A. V., Wei J. T. (2012). Benign prostatic hyperplasia and lower urinary tract symptoms. *New England Journal of Medicine*.

[2] Chughtai B., Forde J. C., Thomas D. D. (2016). Benign prostatic hyperplasia. *Nature Reviews Disease Primers*.

[3] Bushman W. (2009). Etiology, epidemiology, and natural history. *Urologic Clinics of North America*.

[4] Thorpe A., Neal D. (2003). Benign prostatic hyperplasia. *The Lancet*.

[5] Wei J. T., Calhoun E., Jacobsen S. J. (2005). Urologic diseases in America project: benign prostatic hyperplasia. *Journal of Urology*.

[6] Thomas J. A., Tubaro A., Barber N. (2016). A multicenter randomized noninferiority trial comparing GreenLight-XPS laser vaporization of the prostate and transurethral resection of the prostate for the treatment of benign prostatic obstruction: two-yr outcomes of the GOLIATH study. *European Urology*.

[7] Rassweiler J., Teber D., Kuntz R., Hofmann R. (2006). Complications of transurethral resection of the prostate (TURP)-incidence, management, and prevention. *European Urology*.

[8] Cornu J.-N., Ahyai S., Bachmann A. (2015). A systematic review and meta-analysis of functional outcomes and complications following transurethral procedures for lower urinary tract symptoms resulting from benign prostatic obstruction: an update. *European Urology*.

[9] Li S., Zeng X. T., Ruan X. L. (2014). Holmium laser enucleation versus transurethral resection in patients with benign prostate hyperplasia: an updated systematic review with meta-analysis and trial sequential analysis. *PLoS One*.

[10] Mayer E. K., Kroeze S. G. C., Chopra S., Bottle A., Patel A. (2012). Examining the “gold standard”: a comparative critical analysis of three consecutive decades of monopolar transurethral resection of the prostate (TURP) outcomes. *BJU International*.

[11] Varkarakis J., Bartsch G., Horninger W. (2004). Long-term morbidity and mortality of transurethral prostatectomy: a 10-year follow-up. *The Prostate*.

[12] Liu C., Zheng S., Li H., Xu K. (2010). Transurethral enucleation and resection of prostate in patients with benign prostatic hyperplasia by plasma kinetics. *Journal of Urology*.

[13] Zhang K. Y., Xing J. C., Chen B. S. (2011). Bipolar plasmakinetic transurethral resection of the prostate vs. transurethral enucleation and resection of the prostate: pre- and postoperative comparisons of parameters used in assessing benign prostatic enlargement. *Singapore Medical Journal*.

[14] Abou-Taleb A., El-Shaer W., Kandeel W., Gharib T., Elshaer A. (2017). Bipolar plasmakinetic enucleoresection of the prostate: our experience with 245 patients for 3 years of follow-up. *Journal of Endourology*.

[15] Yip S. K., Chan N. H., Chiu P., Lee K. W., Ng C. F. (2011). A randomized controlled trial comparing the efficacy of hybrid bipolar transurethral vaporization and resection of the prostate with bipolar transurethral resection of the prostate. *Journal of Endourology*.

[16] Lusuardi L., Myatt A., Sieberer M., Jeschke S., Zimmermann R., Janetschek G. (2011). Safety and efficacy of eraser laser enucleation of the prostate: preliminary report. *Journal of Urology*.

[17] Zhu L., Chen S., Yang S. (2013). Electrosurgical enucleation versus bipolar transurethral resection for prostates larger than 70 ml: a prospective, randomized trial with 5-year followup. *Journal of Urology*.

[18] Yang Z., Liu T., Wang X. (2016). Comparison of thulium laser enucleation and plasmakinetic resection of the prostate in a randomized prospective trial with 5 year follow-up. *Lasers in Medical Science*.

[19] Netsch C., Becker B., Tiburtius C. (2017). A prospective, randomized trial comparing thulium vapoenucleation with holmium laser enucleation of the prostate for the treatment of symptomatic benign prostatic obstruction: perioperative safety and efficacy. *World Journal of Urology*.

[20] Chiruvella M., Enganti B., Bendigeri M. T., Ghouse S. M., Ragoori D., Reddy P. (2018). Transurethral enucleation with bipolar energy (TUEB):AINU technique and short-term outcomes. *Urology*.

[21] Xu A. B., Luo F., Zou Z. H., Du W., Zhao P. P., Liu C. X. (2016). Application of morcellator in transurethral bipolar plasmakinetic anatomical enucleation of the prostate. *Journal of Southern Medical University*.

[22] Egan K. B. (2016). The epidemiology of benign prostatic hyperplasia associated with lower urinary tract symptoms. *Urologic Clinics of North America*.

[23] Kim E. H., Larson J. A., Andriole G. L. (2016). Management of benign prostatic hyperplasia. *Annual Review of Medicine*.

[24] Thomas D., Chughtai B., Kini M., Te A. (2017). Emerging drugs for the treatment of benign prostatic hyperplasia. *Expert Opinion on Emerging Drugs*.

[25] Masumori N., Kamoto T., Seki N., Homma Y. (2011). Surgical procedures for benign prostatic hyperplasia: a nationwide survey in Japan. *International Journal of Urology*.

[26] Giulianelli R., Gentile B., Albanesi L., Tariciotti P., Mirabile G. (2015). Bipolar button transurethral enucleation of prostate in benign prostate hypertrophy treatment: a new surgical technique. *Urology*.

[27] Gratzke C., Bachmann A., Descazeaud A. (2015). EAU guidelines on the assessment of non-neurogenic male lower urinary tract symptoms including benign prostatic obstruction. *European Urology*.

[28] Naspro R., Gomez S. F., Manica M. (2017). From “gold standard” resection to reproducible “future standard” endoscopic enucleation of the prostate: what we know about anatomical enucleation. *Minerva Urologica e Nefrologica*.

[29] Gacci M., Corona G., Vignozzi L. (2015). Metabolic syndrome and benign prostatic enlargement: a systematic review and meta-analysis. *BJU International*.

[30] Fayad A. S., Elsheikh M. G., Zakaria T. (2015). Holmium laser enucleation of the prostate versus bipolar resection of the prostate: a prospective randomized study. “Pros and cons”. *Urology*.

[31] Bozzini G., Seveso M., Melegari S. (2017). Enucleación con láser de tulio (ThuLEP) frente a resección transuretral de la próstata en solución salina (TURis): un ensayo prospectivo aleatorizado para comparar resultados intra y postoperatorios tempranos. *Actas Urológicas Españolas*.

[32] Lusuardi L., Mitterberger M., Hruby S. (2015). Update on the use of diode laser in the management of benign prostate obstruction in 2014. *World Journal of Urology*.

[33] Luo Y.-H., Shen J.-H., Guan R.-Y., Li H., Wang J. (2014). Plasmakinetic enucleation of the prostate vs plasmakinetic resection of the prostate for benign prostatic hyperplasia: comparison of outcomes according to prostate size in 310 patients. *Urology*.

[34] Zhao Z., Zeng G., Zhong W., Mai Z., Zeng S., Tao X. (2010). A prospective, randomised trial comparing plasmakinetic enucleation to standard transurethral resection of the prostate for symptomatic benign prostatic hyperplasia: three-year follow-up results. *European Urology*.

